# Life, Death, Differentiation, and the Multicellularity of Bacteria

**DOI:** 10.1371/journal.pgen.1000418

**Published:** 2009-03-13

**Authors:** Susan M. Rosenberg

**Affiliations:** Departments of Molecular and Human Genetics, Biochemistry and Molecular Biology, Molecular Virology and Microbiology, and the Dan L. Duncan Cancer Center, Baylor College of Medicine, Houston, Texas, United States of America; Fred Hutchinson Cancer Research Center, United States of America

In recent years, bacterial geneticists and microbiologists have begun moving away from the view that the clonal cell populations they study in the lab are homogeneous lots of identical, autonomous individuals and toward one that was suggested decades ago [1], in which social and even multicellular attributes of bacteria are recognized. Bacterial clones display differentiation, development, cell–cell communication, aging, and even apparent apoptosis, and not just the species with visually appreciable phase variations of surface proteins, spore formation, or variation between swimming and sessile cell types. These features appear to be ubiquitous, applying even to *Escherichia coli*, which has been long regarded as a laboratory model for producing homogeneous cell clones.

Cellular behaviors seem “multicellular” when they appear to confer a group benefit. For example, in many circumstances, bacterial cell–cell communication prevents isolated cells from running cellular programs that work only in groups, like production of light, or attacking a host with toxin proteins [2]. Similarly, many (perhaps all) bacteria differentiate subpopulations that take risks, while the remaining cells stay aloof, hedging the clone's bets—a process called bistability [3],[4]. For example, stress responses instigate the turning up of mutation rate in a small subpopulation of starving *E. coli* cells (reviewed in [5]). This is clearly a dangerous game for most of the cells whose genomes are mutated, but it is one that may provide a shot at producing a rare better-adapted mutant from a clone that is maladapted to its environment, i.e., one that is stressed. Similarly, small cell subpopulations of starving bacteria of many species take up foreign DNA, thus altering their genomes. In *Bacillus subtilis*, the same stress response activates competence for transformation in the subpopulation as increased mutation rate under stress [6], perhaps offsetting the dangerous mutagenic pathway with the ability to regain favorable alleles from (albeit dead) neighbors. Similarly, many bacteria continuously differentiate small subpopulations of temporarily growth-impaired “persister” cells that will lose in a race to colonize new territory rapidly, but can survive a transient blast of antibiotics that will kill their rapidly growing siblings, ensuring some survivors in a clone [3],[4].

Perhaps the most surprising of multicellular-sounding bacterial behaviors is the differentiation of a cell subpopulation slated for programmed cell death. In developing vertebrates, apoptosis kills a layer of eyelid cells so that eyelids may open [7]. In bacteria, many death programs are known but few are understood at this level of “organismal” function (see [8] for an example understood in the program of bacterial sporulation).

In this issue of *PLoS Genetics*, Amitai and colleagues probe the mechanism of programmed cell death caused by the MazF gene in *E. coli* [9]. Many bacteria have death genes as part of toxin/antitoxin (TA) gene modules. These are gene pairs usually co-transcribed in operons. The toxin is a stable, deadly protein but is bound and inactivated by the more labile antitoxin. Cells are safe until some circumstance reduces expression of the operon. This shifts the balance in favor of the stable toxin, causing cell stasis or death. Stressors that induce this shift include various antibiotics, heat shock, starvation, DNA damage, possibly phage infection, and others. Several bactericidal antibiotics appear to kill *E. coli*, because they activate the MazEF system.

Why do cells have TA systems? First discovered in plasmids, TA systems kill cells that lose the plasmid, causing “plasmid addiction”. However, TA pairs are abundant in bacterial chromosomes; *E. coli* has at least five pairs, and *Mycobacterium tuberculosis* may have 60 or more [10]. What are they doing in chromosomes? TA pairs might be selfish genetic elements, apoptosis genes, genome-stabilizing modules that effectively prevent deletion of a chromosomal region [11], genes used for resisting plasmid addiction (by protecting against a plasmid-borne toxin with a chromosomal antitoxin [12]), or inducers of subpopulations of cells in stasis that transiently resist antibiotics (persister cells). The article by Amitai and colleagues offers surprising new details about the mechanism of MazF-mediated killing, and in doing so illuminates what this TA system might be doing for *E. coli*.

**Figure 1:**
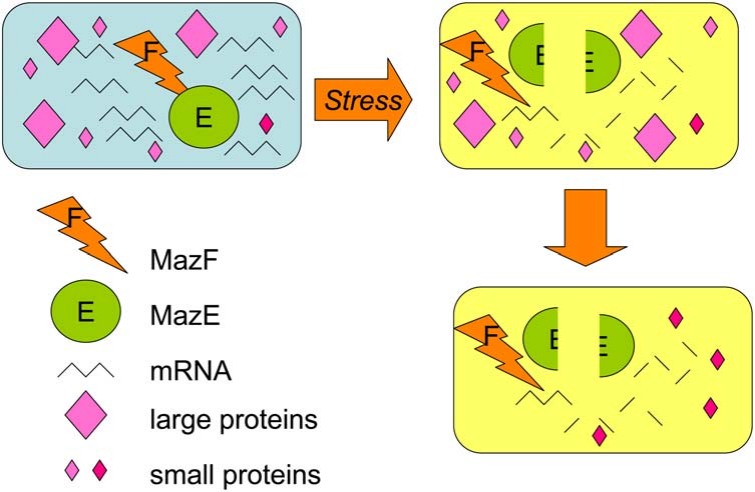
MazF RNase toxin promotes loss of most proteins but selective increase of some small proteins with functions in the MazEF death-and-survival program. When MazF RNase is unmasked by the loss of MazE antitoxin, which binds and inactivates MazF, most cellular mRNAs are degraded, and rapid loss of most proteins occurs. Some mRNAs are protected, and Amitai et al. show that some small (less than 20 kDa) proteins are increased in abundance. Some of these proteins promote the death of most of the cells in the population, whereas others promote the survival of a small cell subpopulation in the MazEF death-and-survival program.

Toxins can kill cells by several routes. Many are RNases, including MazF, which cleaves mRNAs containing the ACA sequence [13]. MazF expression results in a dramatic decrease in cellular protein levels, which was thought to be the cause of MazF-mediated cell death. Amitai et al. revisited the effect of MazF on total cellular protein levels and report the surprising discovery that although the levels of most proteins decrease, levels of some proteins actually increase after MazF overproduction (Figure 1). Amitai et al. displayed the proteomes of the MazF-treated cells on 2D gels and saw that cellular levels of most large proteins (over 20 kDa) decreased while many smaller than 20 kDa increased. They recovered 13 of the up-regulated proteins, identified them with mass spectrometry, then deleted the genes encoding each and tested their effects on MazF-dependent cell death after antibiotic treatment. Surprisingly, all of these proteins contained ACA sequences in their mRNAs, implying that some mechanism, which is not yet understood, protects these mRNAs specifically.

Six of the up-regulated proteins were required for MazF-dependent death, suggesting an active death mechanism. One of the “death proteins,” the ClpP protease, was already known to degrade the MazE antitoxin, acting upstream in the pathway that ultimately unleashes the MazF RNase. It will be interesting to examine whether the rest of the death proteins also allow MazF action, or whether death requires something more than destruction of most of the cell's mRNAs.

Perhaps even more surprisingly, three of the 13 up-regulated proteins, plus another two candidate proteins they tested, are required for survival of a small subpopulation of the cells when most of the cells are killed. This is the first indication that there are “survival proteins” that actively protect a subpopulation when the main population dies. This is reminiscent of bistable populations. In this case one (large) subpopulation is slated for death while a second smaller subpopulation survives, as if there are both death and survival programs activated (in different cells) by MazF. The implication is that the main population is killed so that the subpopulation may survive, supporting the view that MazEF-mediated death serves a multicellular or at least social purpose.

The present study does not distinguish which cells, surviving or dying, make which proteins. Previous work showed that the death program requires cell–cell communication. A secreted pentapeptide, which signals high cell numbers and cellular stress, must be sensed for the program to run [14]. An intriguing question raised here is whether the death proteins are suicide proteins made by the dying cells or assassins sent from those surviving?

Two of the survival proteins protect cells against oxidative damage and can be understood in the context of this group's previous finding that one of the ways that MazF promotes cell death requires oxidative stress—i.e., it can be quenched by any of several means of scavenging reactive oxygen species [15]. These survival proteins are presumably made and used in the surviving cells. How and where the remaining survival proteins work remains to be revealed.

Bacteria lead more coordinated lives than bacterial geneticists initially appreciated. Viewed as groups of individuals, bacteria would seem to be enacting Hamlet- or Macbeth-like tragedies with systems like MazEF. But they may be viewed more usefully, though no less dramatically, as “simply” multicellular.
